# Quality of reporting of trial abstracts needs to be improved: using the CONSORT for abstracts to assess the four leading Chinese medical journals of traditional Chinese medicine

**DOI:** 10.1186/1745-6215-11-75

**Published:** 2010-07-08

**Authors:** Ling Wang, Yulin Li, Jing Li, Mingming Zhang, Lin Xu, Wenming Yuan, Gang Wang, Sally Hopewell

**Affiliations:** 1 Chinese Cochrane Centre, West China Hospital, Sichuan University, Chengdu, Sichuan, China; 2 Department of Nephrology, West China Hospital, Sichuan University, Chengdu, Sichuan, China; 3 Department of Integrated Chinese Medicine and Western Medicine, West China Hospital, Sichuan University, Chengdu, Sichuan, China; 4 NHS R&D Programme, UK Cochrane Centre, Oxford, UK

## Abstract

**Background:**

Due to language limitations, the abstract of journal article may be the only way for people of non-Chinese speaking countries to know about trials in traditional Chinese medicine (TCM). However, little is known about the reporting quality of these trial abstracts. Our study is to assess the reporting quality of abstracts of randomized controlled trials (RCT) published in four leading Chinese medical journals of TCM, and to identify any differences in reporting between the Chinese and English version of the same abstract publication.

**Method:**

Two reviewers hand-searched the Chinese Journal of Integrated Traditional and Western Medicine, the Chinese Journal of Integrative Medicine, the China Journal of Chinese Materia Medica and the Chinese Acupuncture & Moxibustion for all abstracts of RCTs published between 2006 and 2007. Two reviewers independently assessed the reporting quality of the Chinese and English version of all eligible abstracts based on a modified version of the CONSORT for reporting randomised trials in journal and conference abstracts (CONSORT for abstracts).

**Results:**

We identified a total of 345 RCTs of TCM with both a Chinese and English abstract. More than half of Chinese abstracts reported details of the trial participants (68%; 234/345), control group intervention (52%; 179/345), the number of participants randomized (73%; 253/345) and benefits when interpreting the trial results (55%; 190/345). Reporting of methodological quality or key features of trial design and trial results were poor; only 2% (7/345) included details of the trial design, 3% (11/345) defined the primary outcome, 5% (17/345) described the methods of random sequence generation, and only 4% (13/345) reported the number of participants analyzed. No abstracts provided details on allocation concealment and trial registration. The percentage agreement in reporting (between the Chinese and English version of the same abstract) ranged from 84% to 100% across individual checklist item.

**Conclusion:**

The reporting quality of abstracts of RCTs published in these four TCM journals needs to be improved. Since none of the four journals adopted CONSORT for Abstracts, we hope that the introduction and adoption of CONSORT for Abstracts by TCM journals will lead to an improvement in reporting quality.

## Background

Traditional Chinese medicine (TCM) plays an important role in maintaining the health of the Chinese population (especially before the introduction of western medicine into China) and its benefits are gradually being recognized worldwide. A number of randomized controlled trials (RCT) are conducted assessing the efficacy and safety of TCM, the majority of which are published in Chinese in Chinese medical journals with only the abstract being translated into English.

Health professionals often rely on the abstract of a journal article to decide whether to retrieve the full-text. In countries with limited resources to access the full-text, sometimes health care decisions are made based only on the information reported in the abstracts [1,2]. In TCM this problem is exemplified as the English abstract may be the only way for non-Chinese speaking countries to know about RCTs of TCM.

It is therefore important that abstracts of RCTs provide details of the trial participants, interventions, methodology (such as details of randomization, blinding, and intention-to-treat analysis) and the importance of the trial results (including estimates of benefits and harms, and their precision). To date, little is known about the quality of reporting of RCT abstracts in TCM. This study aims to assess and compare the reporting quality of both Chinese and English abstracts of RCTs published in four leading TCM Chinese medical journals using the recent CONSORT for reporting randomised trials in journal and conference abstracts (CONSORT for abstracts) [3].

## Methods

### Selection of journals

We selected four MEDLINE-indexed Chinese medical journals (Chinese Acupuncture & Moxibustion, Chinese Journal of Integrated Traditional and Western Medicine, Chinese Journal of Integrative Medicine, China Journal of Chinese Materia Medica) of TCM with a top ranking impact factor (IF) (ranked as No. 2, 3, 4 and 7) among the 38 journals in the category of Chinese traditional medicine and Chinese materia medica based on the 2007 data for Sci-tech Journal Citation Reports of China (Table 1). We selected these four high impact journals as they are indexed in MEDLINE and each abstract to be reported in English.

**Table 1:** Number of abstracts identified and included from four TCM ^a ^of Chinese medical journals (2006 to 2007)

Journals	**IF **^**b**^	**Identified RCT**^**c**^	Included abstracts
Chinese Acupuncture and Moxibustion	0.437	165	154
Chinese Journal of Integrated Traditional and Western Medicine	0.837	261	158
Chinese Journal of Integrative Medicine	0.598	21	19
China Journal of Chinese Materia Medica	0.634	48	14
Total		495	345

a TCM: traditional Chinese medicine

b Impact factor (IF) comes from «2007 data for sci-tech Journal Citation Reports of China»

c RCT: randomized controlled trial

### Identification of RCTs

Two reviewers (L Wang and YL Li) hand searched the four journals independently. We included all abstracts published between 2006 and 2007 that mentioned "randomization", "randomized allocation" or "randomized" in the abstract or full text. We excluded the abstracts reported as "quasi-randomized controlled trials", "laboratory study", "randomized sampling study", "meta-analysis", or "systematic review". All the eligible abstracts were retrieved from the full text database CNKI (China National Knowledge Infrastructure/Chinese Academic Journals full text Database).

### Assessment of abstracts

We assessed the reporting quality of included Chinese and English abstracts based on criteria outlined in the CONSORT for abstracts [4]. We refined several of the items included in the CONSORT for Abstracts checklist to assess certain items in more detail and to add some unique characteristics relevant to TCM. For example, we expanded "interventions" to "interventions intended for experimental group" and "interventions intended for control group", and expanded the item "blinding" to "specified exactly who was blinded" and "simply described the trials as single blind or double blind". We assessed each item as being "reported" or "not reported" according to whether the author had reported all the contents listed in the refined items or not.

Two reviewers (L Wang and YL Li) assessed each abstract independently after discussing and comprehending the items thoroughly with J Li and MM Zhang. We sought further clarification from S Hopewell if there was any problem with understanding of the CONSORT for Abstracts checklist criteria. A pilot study was performed on 10 RCT abstracts selected at random (both the Chinese and English version of the same abstract) and inter rater agreement was calculated using the kappa statistic. Discrepancies were resolved by discussion. Once we had ensured consistency in the interpretation of the data extraction form we carried out double data extraction on all remaining abstracts. We determined the overall number and proportion (%) of RCT abstracts that reported each of the CONSORT for Abstracts item. We also assessed the consistency in reporting between the Chinese and English version of the same abstract using the kappa statistics. Data were analyzed using Microsoft Excel 2003 and SAS version 9.1.

## Results

We identified 495 RCTs from hand searching the full-text and abstracts of the four leading TCM journals from 2006 to 2007, of which 345 (70%) included both the Chinese and English version of the same abstracts and all were structured abstracts as it is a formal requirement for the four journals. The remaining 150 RCTs were published as short reports with no abstract. Seven RCT reports mentioned "randomized" in the abstract but not in the full text, while 18 mentioned "randomized" in the full text but not in the abstract. Eighty-five papers mentioned "randomized" in the abstract but were judged as "quasi-randomization" after reading the full text. Table 1 provides information on the impact factor for the four journals, the number of RCTs identified and the number of reports with both the Chinese and English version of the same abstracts for each journal. Agreement between the two reviewers was good with a kappa score of greater than 0.75 for each item.

Table 2 presents the criteria used to judge whether the abstract reported the item, and the number and proportion (%) of items reported for both the Chinese and English version of the same abstracts.

**Table 2:** Criteria for assessing the quality of reporting of abstracts of RCTs in TCM

Items	Criteria for judgment	No. of abstracts reporting each item (n = 345)	
		
		Chinese (%)	English (%)
**General items**			
Title	Mentioned randomized or randomly allocation in the title	28(8)	25(7)
Authors	Reported the contact details of corresponding author, e.g. postal address, email	344(99)	344(99)
Trial design	Described as "parallel group", "cluster randomized", "crossover", etc.	7(2)	5(1)
Trial registration	Reported the trial registration number and name of trial register	0(0)	0(0)
Funding	Reported the source of funding for the trial	93(27)	0(0)
**Methods**			
Participants	Reported the details of the participants (included the demography, diagnosis, TCM syndrome)	234(68)	239(69)
Interventions	Reported the details of the interventions for experimental group (included denomination, usage and course of treatment)	163(47)	152(44)
	Reported the details of the interventions for control group (included denomination, usage and course of treatment)	179(52)	168(49)
Objective	Described as "to compare interventions(A) and (B) in patients with condition(C)"	48(14)	44(13)
Outcome	Defined the primary outcome	11(3)	10(3)
Randomization	Reported the method of random sequence generation	17(5)	14(4)
	Mentioned the allocation concealment (such as central randomization by telephone)	0(0)	0(0)
Blinding	Specified exactly who was blinded	1(0.3)	0(0)
	Simply mentioned the trial as "single blind", or "double blind"	39(11)	39(11)
**Results**			
Numbers randomized	Reported the number of participants randomized to each group	253(73)	253(73)
Numbers analyzed	Reported the number of participants included in analysis for each group	13(4)	12(4)
Outcomes	Reported the result for each group (if the primary outcome not defined, reported the result of one listed outcome)	195(57)	190(55)
	Reported effect size, precision for the difference between groups	2(1)	2(1)
Harms	Reported important adverse events or side effects	50(15)	48(14)
**Conclusions**			
	Clearly stated the benefits and harms when interpreting the results	4(1)	3(1)
	Only stated the benefits when interpreting the results	190(55)	190(55)

### Reporting quality of Chinese RCT abstracts

#### General items

Only 8% (28/345) abstracts could be judged as RCTs from their titles, other abstracts were most described as "clinical trial", "comparison research", "effect comparison", or "effect observation". Only one (1/345) abstract did not report the author's addresses, seven abstracts reported the trial design as "parallel comparison RCTs", no abstract reported details of trial registration and only 27% (93/345) reported the source of funding.

#### Trial methodology

A total of 68% (234/345) of abstracts reported details of participants included in the trial; the remainder simply used the term "patient". Forty-seven percent (163/345) of abstracts reported details of the experimental intervention and 52% (179/345) the control intervention. Forty-two percent (144/345) of abstracts reported the name of the experimental intervention and 27% (92/345) the control intervention, 11% (38/345) described the experimental intervention as "Chinese medicine" and 21% (74/345) of the control intervention as "western medicine". Only 14% (48/345) of abstracts reported the study objective, 5% (17/345) the method of random sequence generation; no abstracts mentioned details of allocation concealment. A total of 11% (39/345) mentioned the trial as "single blind" or "double blind"; only one specified exactly who was blinded.

#### Trial results

Seventy-three percent (253/345) of abstracts reported the number of participants randomized to each group, however, only 4% (13/345) reported the number of participants included in the analysis. Fifty-seven percent (195/345) of abstracts reported the result for each group and 44% (150/345) reported a P value for the between-group comparison, however only two abstracts reported the effect size and its precision. Fifteen percent (50/345) of abstracts mentioned adverse events or side effects of the treatment.

#### Trial conclusion

Four abstracts clearly stated the benefits and harms of the results when interpreting the clinical application, with 55% (190/345) of abstracts reporting only the benefits.

#### Comparison of the Chinese and English of the same RCT abstracts

The proportion of abstracts reporting each checklist item was almost the same between the Chinese and corresponding English abstracts (Table 3). The percentage agreement in reporting (between the Chinese and English version of the same abstracts) ranged from 84% to 100% across individual checklist item. The median of kappa statistic for each item was 0.86 (0.64-1.00). The items where there was most disparity between the Chinese and English abstracts were details of the trial participants and the number of participants randomized.

**Table 3:** Consistency in reporting between Chinese and English abstracts

Items	No. of consistent abstracts (%)	Kappa[95%CI]
Title	338(98%)	0.86[0.75 to 0.96]
Author	344(100%)	1.00[1.00 to 1.00]
Trial design	343(99%)	0.85[0.65 to 1.00]
Participants	291(84%)	0.64[0.55 to 0.72]
Interventions	316(92%)	0.86[0.80 to 0.90]
Objective	328(95%)	0.76[0.66 to 0.86]
Outcome	343(99%)	0.95[0.85 to 1.00]
Randomization	338(98%)	0.76[0.59 to 0.93]
Blinding	345(100%)	1.00[1.00 to 1.00]
Numbers randomized	309(90%)	0.73[0.65 to 0.81]
Numbers analysis	344(100%)	0.91[0.79 to 1.00]
Outcome	326(95%)	0.89[0.84 to 0.94]
Harms	335(97%)	0.88[0.80 to 0.95]
Conclusions	316(92%)	0.99[0.98 to 1.00]

## Discussion

Our study identified 345 of RCTs published in four leading Chinese TCM journals with both the Chinese and English version of the same abstracts between 2006 and 2007. Based on the CONSORT for Abstract checklist criteria, the quality of reporting of trial abstracts in these journals was far from satisfactory. Except for details of the trial authors, participants, the intervention for control group, and the number of participants randomized in the trial, all other items were reported in less than half of the abstract reports. Reporting of methodological quality or key features of trial design and trial results was even worse. Less than 5% of the included abstracts fully or correctly reported relevant items including details of the trial design, clearly defining the primary outcome, the method of random sequence generation and allocation concealment, specifying details on who was blinded, the number of participant analyzed in each arm of the trial, and the estimated effect size and its precision for the primary outcome.

Our study has several strengths. We hand searched four high impact TCM medical journals to ensure that all RCTs were identified. In addition, all items in modified version of the CONSORT for Abstracts checklist were carefully discussed and understood within the research team. The identification of RCTs, eligibility decision and data extraction were conducted in duplicate. However, our study has limitations. We did not take a random sample of all RCT published in TCM medical journals and therefore our findings may not reflect the whole picture of reporting quality of trial abstracts in the TCM field.

Our findings are consistent with or worse than previous studies that have assessed the quality of reporting of journals RCT abstracts and conference RCT abstracts. Previous work has shown that abstracts frequently under report key features of study design [1,2,5] and omit important results. In the mid-1990s, Li and Hu reported that the quality of abstracts in Chinese medical journals was poor [6,7]. The problem is particularly apparently when trying to assess details of methodological quality. A recent study evaluating 227 RCT abstracts published in four high impact general medical journals in 2006 found that only one abstract reported details of allocation concealment, 23% reported the use of intention-to-treat analysis, 14% reported loss of follow-up, and 9% reported details of blinding [8]. A further study evaluating RCT abstracts presented at the American Society of Clinical Oncology Conference and found no abstracts reported allocation concealment, 16% reported on blinding, and only 14% reported intention-to-analysis [9].

Possible explanations for our findings of poor reporting include poor reporting of RCT full-text articles in China, when an item isn't reported in the full publication, it may also be missing from the abstract. A study evaluating the reporting quality of 142 RCT full-text articles published in five leading Chinese medical journals found that only 27% described the method to generate the randomized sequence, 4% had adequate allocation concealment, and only 17% mentioned blinding [10]; these findings are supported by others in the field of TCM [11,12].

Lack of detailed requirements for structured abstracts in journal's "Instruction for Authors" in China is also a problem since none of the journals included in our study mentioned CONSORT for Abstracts in their Instructions to Authors. Some studies have proved that journal adoption of structured abstracts can improve the reporting of abstracts [13-17]. Although most of the Chinese medical journals have adopted four-heading structured abstracts (objectives, methods, results and conclusion), the structured format alone is insufficient to guide authors regarding their content for reporting RCTs.

Finally, the word limitation of an abstract restricts the amount of detail an author is able to provide. Most Chinese medical journals have abstract word restrictions, usually within 150-500 Chinese words, making it difficult for authors to comprehensively report trial methods and results, especially when authors are not aware the importance of research methodology and the way to correctly present the clinical relevance of research results. The word limit for the journals we assessed is 150-250 Chinese words and 150-400 English words.

## Conclusion

In summary, we found that the quality of reporting of trial abstracts in these four leading TCM medical journals is far from satisfactory, especially with respect to details of the trial methodology. Many studies have proved that adoption of the CONSORT Statement has demonstrated benefits in improving the reporting quality of RCT of full-text articles [18], and we hope that it will do the same with the introduction of CONSORT for Abstracts in Chinese medical journals of TCM field.

## Competing interests

This research is supported by a project of the Cochrane Collaboration entitled "Making Cochrane review more accessible to policy makers", but no funding for the publication. Dr Sally Hopewell is a lead author on the CONSORT extension for reporting abstracts of randomized trials.

## Authors' contributions

LW and YL participated in the design of the study, carried out the hand searching of journals, assessment of the abstracts and drafting the manuscript. JL, MZ and SH conceived the study, interpreted the data and revised the manuscript. LX and WY participated in the design of the study and performed the data analysis. GW participated in the design of the study, selection of journals and interpretation of the data. All authors read and approved the final manuscript.

## References

[B1] Barry H Ebell M Shaughnessy A Slawson D Nietzke F Family physicians' use of medical abstracts to guide decision making: style or substance? The Journal of the American Board of Family Medicine 2001 14 437 11757886

[B2] Chen Y Du L He D Reading Habits of Authors in the Chinese Journal of Evidence-Based Medicine: A Questionnaire Survey Chinese Journal of Evidence-Based Medicine 2008 8 312 314

[B3] Hopewell S Clarke M Moher D Wager E Middleton P Altman DG Schulz KF CONSORT for reporting randomised trials in journal and conference abstracts Lancet 2008 371 281 283 10.1016/S0140-6736(07)61835-2 18221781

[B4] Hopewell S Clarke M Moher D Wager E Middleton P Altman DG Schulz KF CONSORT for reporting randomized controlled trials in journal and conference abstracts: explanation and elaboration PLoS Med 2008 5 e20 10.1371/journal.pmed.0050020 18215107 PMC2211558

[B5] Narine L Yee DS Einarson TR Ilersich AL Quality of abstracts of original research articles in CMAJ in 1989 CMAJ 1991 144 449 453 1993292 PMC1452817

[B6] Hu BL Quality of abstracts analysis of the Chinese Journal of Arteriosclerosis from 1994 to 1996 Chin J Arteriosclerosis 1997 5 85 90

[B7] Li AR Problems of abstracts published in 1993 in *Chinese Journal of Internal Medicine* Zhonghua Nei Ke Za Zhi 1995 34 65 66

[B8] Berwanger O Ribeiro RA Finkelsztejn A Watanabe M Suzumura EA Duncan BB Devereaux PJ Cook D The quality of reporting of trial abstracts is suboptimal: Survey of major general medical journals J Clin Epidemiol 2008 19010643 10.1016/j.jclinepi.2008.05.013

[B9] Hopewell S Clarke M Abstracts presented at the American Society of Clinical Oncology conference: how completely are trials reported? Clin Trials 2005 2 265 268 10.1191/1740774505cn091oa 16279150

[B10] Xu L Li J Zhang M Ai C Wang L Chinese authors do need CONSORT: Reporting quality assessment for five leading Chinese medical journals Contemporary Clinical Trials 2008 29 727 731 10.1016/j.cct.2008.05.003 18579449

[B11] Mao B Wang G Fan T Chen X Liu J Wang L Chang J Ma J Guo J Fu J Li T Assessing the Quality of Reporting of Randomized Controlled Trials in Traditional Chinese Medicine Chinese Journal of Evidece-based Medicine 2007 7 880 887

[B12] Xiao-li Zhang Jing Li Ming-ming Zhang Wen-ming Yuan Assessing the Reporting Quality of Randomized Controlled Trials on Acupuncture for Acute Ischemic Stroke Using the CONSORT Statement and STRICTA Chinese Journal of Evidence-Based Medicine 2006 6 586 590

[B13] Hopewell S Eisinga A Clarke M Better reporting of randomized trials in biomedical journal and conference abstracts Journal of Information Science 2008 34 162 10.1177/0165551507080415

[B14] Taddio A Pain T Fassos FF Boon H Ilersich AL Einarson TR Quality of nonstructured and structured abstracts of original research articles in the British Medical Journal, the Canadian Medical Association Journal and the Journal of the American Medical Association CMAJ 1994 150 1611 1615 8174031 PMC1336964

[B15] Narine L Yee DS Einarson TR Ilersich AL Quality of abstracts of original research articles in CMAJ in 1989 CMAJ 1991 144 449 453 1993292 PMC1452817

[B16] Trakas K Addis A Kruk D Buczek Y Iskedjian M Einarson TR Quality assessment of pharmacoeconomic abstracts of original research articles in selected journals Ann Pharmacother 1997 31 423 428 9101002 10.1177/106002809703100406

[B17] Dupuy A Khosrotehrani K Lebbe C Rybojad M Morel P Quality of abstracts in 3 clinical dermatology journals Arch Dermatol 2003 139 589 593 10.1001/archderm.139.5.589 12756095

[B18] Moher D Jones A LePage L Use of the CONSORT Statement and quality of reports of randomized trials: a comparative before-and after evaluation JAMA 2001 285 1992 1995 10.1001/jama.285.15.1992 11308436

